# Correlating Magnetic Hyperthermia and Magnetic Resonance Imaging Contrast Performance of Cubic Iron Oxide Nanoparticles with Crystal Structural Integrity

**DOI:** 10.1021/acs.chemmater.2c00708

**Published:** 2022-08-11

**Authors:** Sameer D. Shingte, Abhijit H. Phakatkar, Eoin McKiernan, Karina Nigoghossian, Steven Ferguson, Reza Shahbazian-Yassar, Dermot F. Brougham

**Affiliations:** † School of Chemistry, University College Dublin, Dublin 4, Ireland; ‡ Department of Biomedical Engineering, University of Illinois at Chicago, Chicago, Illinois 60607, United States; § School of Chemical and Bioprocess Engineering, University College Dublin, Dublin 4, Ireland; ∥ Department of Mechanical and Industrial Engineering, University of Illinois at Chicago, Chicago, Illinois 60607-7042, United States

## Abstract

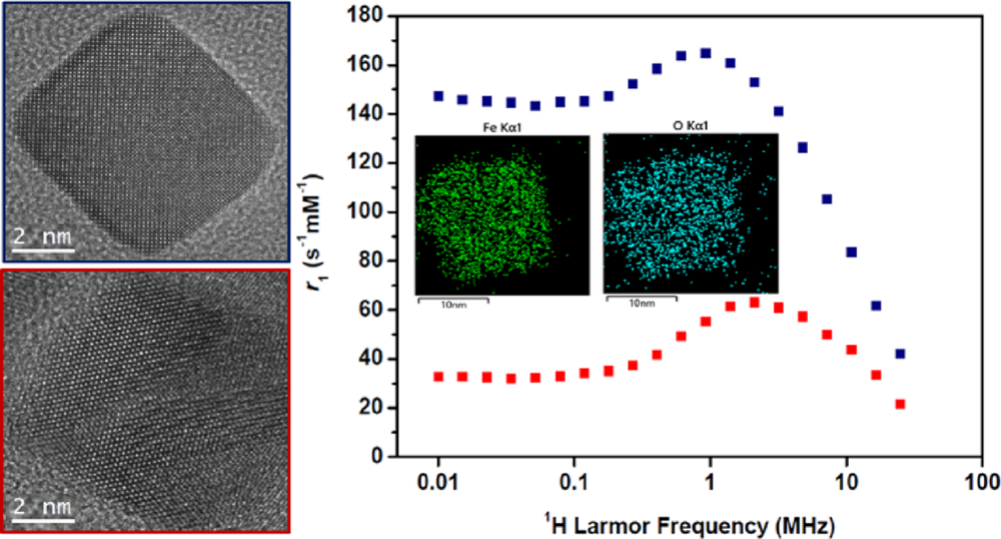

Magnetic iron oxide nanoparticles have multiple biomedical applications in AC-field hyperthermia and magnetic resonance imaging (MRI) contrast enhancement. Here, two cubic particle suspensions are analyzed in detail, one suspension displayed strong magnetic heating and MRI contrast efficacies, while the other responded weakly. This is despite them having almost identical size, morphology, and colloidal dispersion. Aberration-corrected scanning transmission electron microscopy, electron energy loss spectroscopy, and high-resolution transmission electron microscopy analysis confirmed that the spinel phase Fe_3_O_4_ was present in both samples and identified prominent crystal lattice defects for the weakly responding one. These are interpreted as frustrating the orientation of the moment within the cubic crystals. The relationship between crystal integrity and the moment magnitude and dynamics is elucidated for the case of fully dispersed single nanocubes, and its connection with the emergent hyperthermia and MRI contrast responses is established.

## Introduction

Magnetic iron oxide nanoparticles (MNPs) are under intensive scrutiny for biomedical applications due to their responses to: (i) static homogeneous DC-magnetic fields in which the strong moments can provide contrast enhancement for magnetic resonance imaging (MRI);^1^ (ii) DC-magnetic field gradients which provide magnetophoretic particle motion;^2,3^ and (iii) AC-fields in the kilohertz range which generate localized heating applicable for cancer ablation^4^ or thermally triggered drug release.^5^ Next-generation magnetic nanomaterials are expected to involve combinations of these functions.^6,7^

However, while the understanding of how the internal particle structure determines the strength and dynamics of the moments is quite advanced,^8^ the impact of structure on both the AC-field heating and MRI contrast efficacies, i.e., the specific absorption rate (SAR) and the relaxivities (*r*_1_ and *r*_2_), is missing, and this limits development of the dual magnetic function. Significant practical challenges also remain to reproducibly process MNPs into stable suspensions at the application scale with appropriate crystallinity, size, and colloidal dispersion to provide strong moments with appropriate internal dynamics to control the responses.^9^ It is also well known that repeated syntheses using the favored thermal decomposition approaches can produce samples with rather different magnetic properties, reflecting high sensitivity of the final nanocrystals to minor differences/fluctuations in particle nucleation and growth during the process.

Suspensions of spherical MNPs usually show a SAR of ≤100 W g^–1^ under typical field conditions. The field strength and frequency dependence of the SAR can be significant and should be borne in mind when making performance comparisons;^10^ however, most measurements are in the range ν_AC_ 200–700 kHz and *I*_Ac_ 5–20 kA m^–1^.^11,12^ High SAR values have been reported, usually due to more open room temperature AC-field hysteresis loops under the field conditions used, arising from increased magnetocrystalline anisotropy energy, Δ*E*_anis_. This can be generated through Co- or Mn-doping of the oxide;^13^ through formation of ordered (low particle number) chains,^14^ which can also be extracted from magnetosomes;^15^ or through particle shape anisotropy. All three approaches are synthetically challenging and difficult to upscale, with shape control perhaps the most amenable to optimization.

In this regard cubic MNPs have shown significant promise. Guardia et al. described SAR dependence on size, demonstrating a higher SAR of ca. 2300 W g^–1^ (at 700 kHz and 24 kA m^–1^) for 19 nm than for 12, 25, or 38 nm cubes.^16^ Nogues and co-workers showed an ∼74% increase in the SAR to ca. 192 W g^–1^ and ca. 40% enhancement in the spin–spin relaxivity, *r*_2_, on increasing size from 13 to 19 nm.^12^ Nemati et al. reported a threefold higher SAR under similar field conditions for cubic as compared to spherical MNPs of the same volume,^17^ and similarly, Martinez-Boubeta et al. showed that 20 nm nanocubes exhibited 20% higher SAR compared to the spherical counterparts of the same diameter.^14^ Significant issues emerge including: (i) the complexity of size control in these systems. Much of this literature details efforts to control average size, polydispersity, shape, crystallinity, and magnetic properties; (ii) again, few details are provided on batch-to-batch variability; and (iii) the dual, hyperthermic, and MRI functionalities have not been rationalized in the context of nanocrystal microstructural analysis.

Pellegrino and co-workers correlated low SAR (in toluene and water) for spherical MNPs in the size (*d*_TEM_) range from 10 to 18 nm with crystal structure and lattice distortions.^11^ High-resolution transmission electron microscopy (HR-TEM) coupled with peak pair analysis was employed for poorly responsive spherical 18 nm MNPs only, from which it was suggested that lower heating efficacies are associated with strained regions (magnetically frustrated layers) within the lattice that adversely affect dynamic alignment of the moments.

Here, a systematic study was performed on two heptane suspensions of cubic MNPs prepared in apparently identical fashion which showed very similar particle size, morphology, and critically, full particle dispersion. The final suspensions, unsurprisingly for thermal decomposition synthesis, differed strikingly in their SAR and spin–lattice relaxivity, *r*_1_. This difference enabled the first comparative assessment of the role of crystal structure integrity on the magnetic properties. Aberration-corrected scanning transmission electron microscopy (STEM) and electron energy loss spectroscopy (EELS) elemental analysis of cubic MNPs demonstrated indistinguishable chemical composition and phase. Microstructural analysis revealed crystal defects for the weakly responsive materials which are interpreted as frustrating moment reorientation.

There have been reports on the relaxivity of MNPs of different shapes, including spheres,^18^ cubes,^12^ and nanoflowers,^19^ and structure–relaxivity relationships in MNPs for MR imaging have been recently reviewed.^20^ For particles in the size range of this study, large moments generate high *r*_2_, and so dephasing (local image suppression due to rapid ^1^H spin–spin relaxation) overwhelms any *T*_1_-weighting effects (local image enhancement due to rapid ^1^H spin–lattice relaxation). The more complex field shape generated by the cubic particles has been reported to exacerbate this effect.^20^ For these reasons, *r*_1_ and *r*_2_ values in the clinical MRI frequency range are usually reported. In contrast, in this study, we exploit measurement of the ^1^H Larmor frequency dependence of *r*_1_. The low-frequency relaxivity in particular reveals changes in the moment dynamics, arising from the presence of defects, which largely determine the SAR achieved. To the best of our knowledge, this is the first report establishing how crystallinity influences the moment magnitude and dynamics which determine the hyperthermic and contrast efficacies for fully dispersed single crystals.

## Experimental Section

### Synthesis

Iron oleate (FeOl) is one of the key precursors to synthesize iron oxide MNPs having a cubic morphology. MNP formation using FeOl involves two main stages; precursor synthesis and then MNP synthesis. To synthesize the precursor, Fe(III) chloride hexahydrate (10.8 g, 40 mmol) and sodium oleate (36.5 g, 120 mmol) were added into a mixture of 80 mL of ethanol, 60 mL of deionized water, and 140 mL of heptane in a 500 mL three-necked round-bottomed flask and stirred vigorously overnight (minimum 12 h). The next day, the reaction mixture was refluxed (at ca. 70 °C) for 7 h under nitrogen flow. Once the reaction mixture cooled down, it was transferred to a separating funnel. The mixture separates into two distinct phases, the lower (aqueous phase) was removed and disposed of. The upper organic phase that contains FeOl was washed with hot deionized water (∼80 °C) and the bottom aqueous phase was discarded repeatedly. The main purpose of this washing was to get rid of salts and aqueous-based impurities from the organic phase. This washing step was repeated 60 times. MNPs synthesized with unwashed FeOl showed two distinct morphologies: cubes and relatively smaller (∼12.3 nm) nanospheres. The fraction of cubes increased with the number of washes, but a noncubic fraction remained at 10 times washed. With 30 and 60 times washed precursors nearly perfectly cubic-shaped MNPs were formed. Afterward, the organic phase was dried over magnesium sulfate and then concentrated by a rotary evaporator. Thereafter, a viscous brown liquid was obtained which was stored under ambient conditions under nitrogen.

The cubic MNP synthesis is based on the protocol given by Shavel et al.;^21^ 1.5 g of iron oleate, 250 mg of sodium oleate, 543 μL of oleic acid, and 20 mL of squalene (CAS: 111-02-4, C_30_H_50_) were added to a 100 mL round-bottom flask. First, the reaction mixture was degassed for 2 h at 120 °C with vigorous nitrogen flow. This step eliminates traces of moisture and dissolved oxygen in the reaction mixture. Then, the mixture was kept at reflux for 2 h. After this period, and once the reaction mixture had cooled to room temperature, the MNPs were precipitated by ethanol and any of the original solvent and other impurities were removed by centrifugation (SIGMA Laborzentrifugen GmbH) operated at 8000 rpm for 10 min. This process was repeated three times by sequential dispersal in heptane/ethanol precipitation added in proportion of 1:4 by volume, after which the MNPs were stabilized in heptane.

### AC-Field Hyperthermia

The efficacy of MNPs is quantified using the SAR, which has units of W g^–1^ and which quantifies the ability of the material to convert magnetic energy provided by an AC-field into heat energy.^17^ In this study, a calorimetric approach was used in which an AC-field was applied and the suspension temperature was continuously monitored. A NanoTherics NAN201003 magneTherm AC-field generator (NanoTherics Ltd.; Newcastle-under-Lyme, United Kingdom) was used throughout. Typically, 1.0 mL of MNP suspension (∼20 mm height), having Fe concentration ranging between 30 and 120 mM, was transferred to a “cylindrical” eppendorf tube and placed in a thermally insulating polystyrene sample holder to provide close-to-adiabatic conditions. The sample temperature was measured by using a nonmetallic temperature sensor (Opsens Ltd., Canada). The probe was typically placed at a 15 mm depth from the suspension surface. The temperature of the sample was equilibrated in the instrument before the AC-field was applied. All measurements were undertaken at ν_AC_ 535 kHz and *I*_Ac_ 16.0 kA m^–1^. The temperature increase over time on application of the field was recorded and the SAR value was calculated using eq 1:
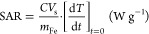
1

where *C* is the volumetric specific heat capacity of the solvent (J mL^–1^ °C^–1^), *V*_s_ is the sample volume (mL), *m*_Fe_ is the mass of Fe in the suspension (g), and  is the initial slope of the curve (°C s^–1^) extracted here as the coefficient of the linear term in a fourth-order polynomial fit. The error associated with the individual SAR measurements was ∼5% for high heating MNPs. The iron content was measured following digestion^22^ using flame atomic absorption spectroscopy.

### Atomic Absorption Spectrometry

For AAS analysis a Varian SpectrAA 55B atomic absorption spectrometer equipped with a single slit burner was used. A Fe cathode lamp operating at a wavelength of 248.3 nm was used as the light source and the flame was maintained with a mixture of air and acetylene. For a typical measurement, standards of Fe concentration between 0.1 and 7.0 ppm were prepared by diluting a Fe standard solution of known concentration 1000 ppm from Sigma-Aldrich (CAS: 10421484) in 1 M HNO_3_. The absorbances were measured for these standards to obtain a calibration curve. Aliquots of known volume were taken from the MNP suspensions diluted appropriately to ensure that the Fe concentration would fall within the calibrated range. These aliquots were digested in HCl (12 M, 1.5 mL) for 2 h prior dilution with 1 M HNO_3_ in a volumetric flask.

### Dynamic Light Scattering

Colloidal properties were measured by dynamic light scattering (DLS) using a Malvern NanoZS (Malvern Instruments, Malvern, UK) instrument. This instrument uses a 3 mW He–Ne laser working at a wavelength of 633 nm with the scattered light detected at 173°. For a typical DLS measurement, around 1 mL of suspension of 0.5–5 mM Fe concentration was used. Measurements were carried out at 25 °C. The Z-average size and polydispersity index (PDI) were obtained from cumulant analysis through the analysis of the correlation functions using Dispersion Technology software (v. 4.10, Malvern Instruments; Worcestershire, UK). All distributions were unimodal, the PDI values were low, and there were no longer time features in the correlation functions; hence, the *Z*-average size is used throughout as a measure of the average hydrodynamic size, *d*_hyd_.

### Fast-Field-Cycling Nuclear Magnetic Resonance (FFC-NMR)

The spin–lattice relaxivity (*r*_1_, expressed in mM^–1^ s^–1^) is defined as the ^1^H spin–lattice relaxation rate enhancement per millimolar of Fe in the suspensions and is calculated by using following equation:
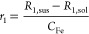
2where *R*_1, sus_ and *R*_1, sol_ are the spin–lattice relaxation rates (units, s^–1^) of the MNP suspension and pure solvent, respectively, and *C*_Fe_ is the mM Fe concentration of the suspension. Hence, the numerator in eq 2 is the relaxation enhancement. The dependence of *r*_1_ on the applied field (and hence frequency, ν_L_ = *γB*_0_/2π, where γ is the gyromagnetic ratio for ^1^H or 2.6575 × 10^8^ rad T^–1^ s^–1^) for the MNP suspensions was measured by fast-field-cycling NMR relaxometry (FFC-NMR) using a Stelar Spinmaster FFC2000. This instrument employs a fast-cycling electromagnet to vary the applied field across a range corresponding to the ^1^H Larmor frequencies from 0.01 to 40 MHz. The system operated at a measurement frequency of 16.3 MHz for ^1^H, with a 90° pulse of 7 μs using standard pulse sequences.^23^ The temperature of the suspensions was maintained at 25 °C using a thermostat airflow system.

### Low-Magnification Transmission Electron Microscopy

MNPs were characterized at low magnification using a FEI Tecnai G2 20 TWIN 200 kV TEM microscope. Typical sample preparation for imaging involved spotting ∼3 μL of an MNP suspension of Fe concentration 2–5 mM onto a formvar-coated copper grid and allowing natural drying. The images were analyzed using ImageJ software to measure *n* ≥ 100 particle sizes, which were averaged to calculate, *d*_TEM_.

### High-Resolution Transmission Electron Microscopy

An aberration-corrected JEOL ARM200CF electron microscope in the TEM mode operating at 200 kV was utilized to acquire atomic resolution (HR-TEM) micrographs of cubic iron oxide MNPs. A beam current condition of 15 μA was maintained throughout.

### Selected Area Electron Diffraction

Selected area electron diffraction (SAED) bulk crystal structure analysis of MNPs was performed using a JEOL JEM-3010 transmission electron microscope operating at 300 keV. A camera length of 20 cm was selected for data acquisition.

### X-ray Diffraction

MNP suspension in heptane were dried for X-ray diffraction (XRD) characterization. The analysis was performed using an X-ray diffractometer (Siemens D500), with data acquired in the 25–70° 2θ range, with an exposure time of 7 s and accelerating voltage and current 40 kV and 30 mA, respectively.

### STEM-Energy-Dispersive X-ray Spectroscopy and STEM-EELS

To evaluate the elemental distribution of the synthesized iron oxide nanoparticles, STEM-EDS was performed using an aberration-corrected JEOL ARM200CF 200 kV electron microscope in the STEM mode. Elemental mapping was performed using an Oxford EDS system in the drift corrector acquisition mode. Qualitative chemical analysis of MNPs was carried out using STEM-EELS analysis. A GATAN annular dark-field (ADF) STEM detector with a collection semiangle of 53.4 mrad and 0.3 eV/ch energy dispersion with a step size of 1 nm was utilized for the dual-EELS spectral acquisition.

## Results and Discussion

**Figure 1:**
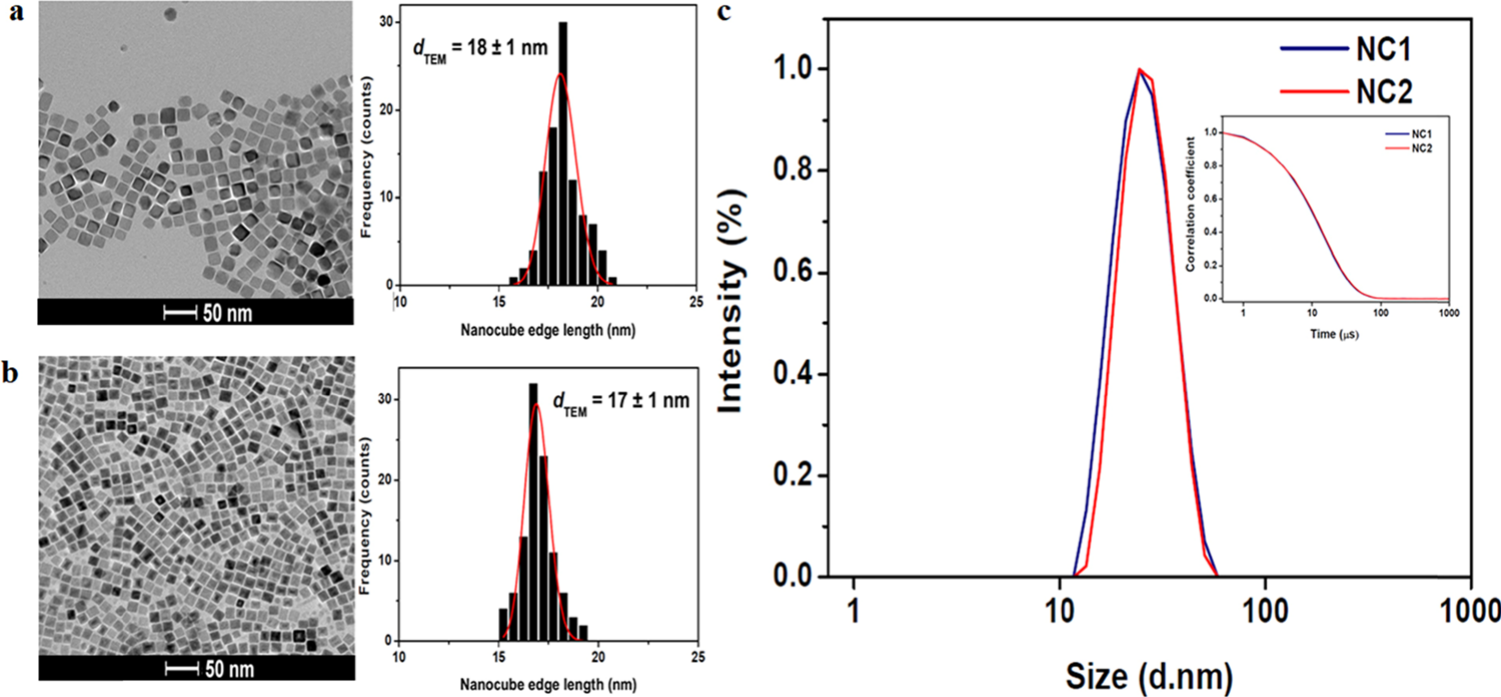
Primary magnetic nanocube characterization. Representative TEM images of cubic MNPs along with their respective size histograms for (a) NC1 and (b) NC2. A lognormal distribution was confirmed by fitting the *d*_TEM_ values given are the sample (*n* = 100) averages. (c) DLS characterization (intensity size distribution) of the same samples in heptane at 25 °C. Inset: correlation functions.

Two stable suspensions of cubic MNPs in heptane were selected from a large number of otherwise identical suspensions prepared using the thermal decomposition approach described in the Experimental Section. There were no apparent differences in the progress of the reaction in any case. The samples selected showed strong and weak hyperthermic responses, see below. TEM analysis Figure 1a,b demonstrated the expected morphological similarities between the two samples. In both cases square shapes are apparent in the images; average nanocube sizes, *d*_TEM_, of 18 ± 1 and 17 ± 1 nm were measured for samples NC1 and NC2, respectively both with an average aspect ratio of 1.0 ± 0.1. The distributions are unimodal for both samples with almost no smaller particles or aggregates evident. As our focus was on the effect of crystal integrity on the magnetic properties of the suspensions, cubic MNPs surface-stabilized with oleic acid in the organic solvent heptane were prepared to ensure full particle dispersion. This approach has been adopted by others.^12,21,24^ The colloidal properties were assessed by DLS (see Figure 1c) in the typical Fe concentration range of ∼0.5–5.0 mM also used for FFC-NMR (see below). The *d*_hyd_ values were 24.5 nm (PDI 0.08) and 25.2 nm (0.05), for NC1 and NC2, respectively. The correlation functions were completely superimposable, demonstrating effectively indistinguishable hydrodynamic sizes.

**Figure 2:**
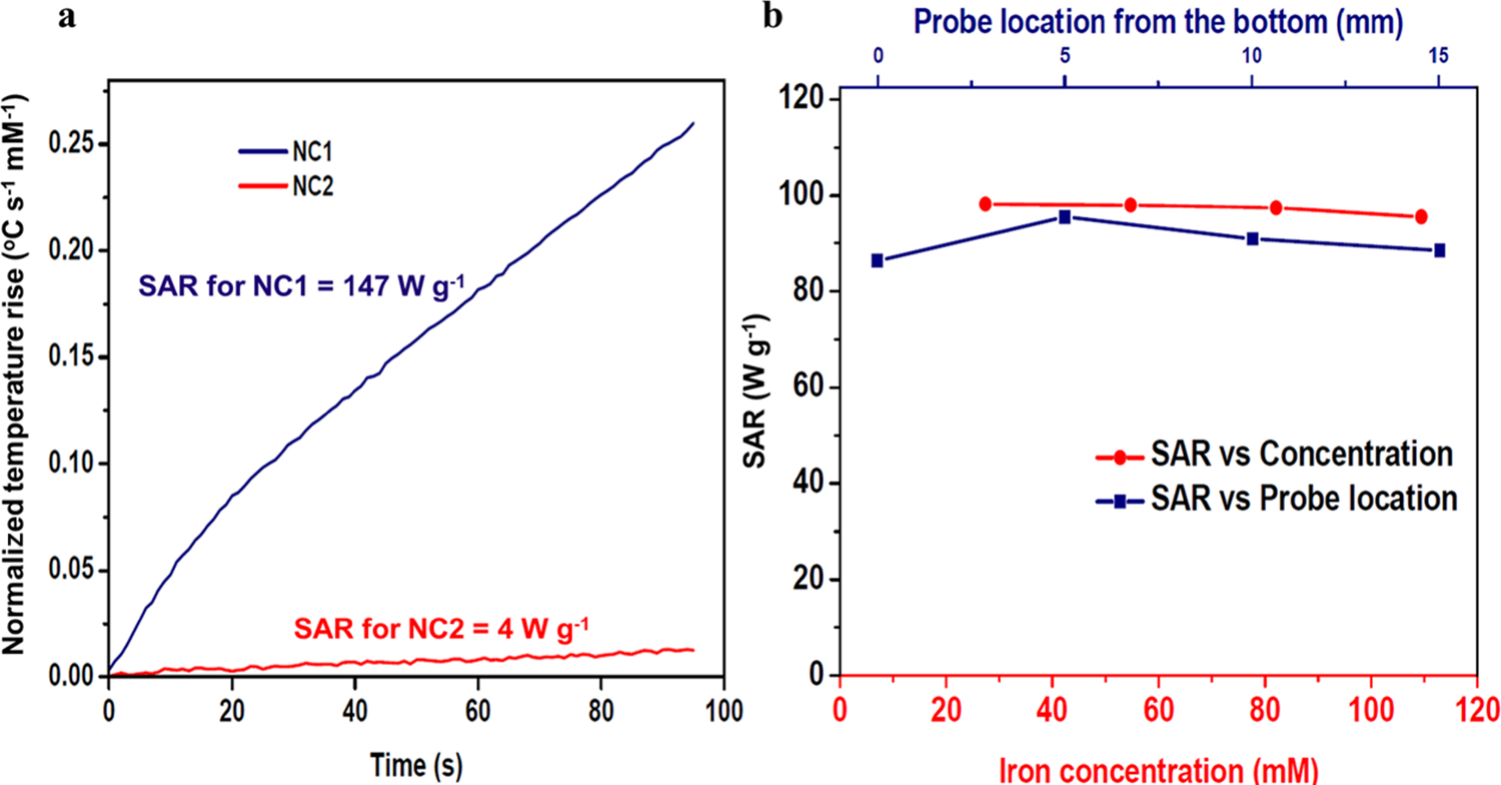
AC-field hyperthermic efficacy of cubic MNP suspensions in heptane at *ν*_AC_ 535 kHz and *I*_AC_ 16.0 kA m^–1^. (a) AC-field-induced temperature increases for suspensions NC1 and NC2. (b) SAR values of a representative cubic MNP suspension (NC3, *d*_TEM_ 18 ± 1 nm) as a function of: (i) Fe concentration between 27 and 109 mM at probe location 5 mm from the bottom of the sample holder and (ii) probe location between 0 to 15 mm for a suspension of Fe concentration 109 mM.

The AC-field hyperthermic efficacy of the suspensions was measured at ν_AC_ 535 kHz and *I*_Ac_ 16.0 kA m^–1^, Figure 2a, with SAR values of 147 and 4 W g^–1^ obtained at Fe concentrations of 55 and 43 mM for NC1 and NC2, respectively. It is well known that magnetic properties of MNP suspensions are largely determined by particle size, shape,^7^ and dispersion; hence, this great difference in the SAR is initially surprising.

A collective particle scenario (arising from reversible aggregation) which increased the SAR has been described for suspensions in this Fe concentration range.^25^ To confirm the validity of the measured values, the concentration dependence of the SAR was subsequently measured for a different representative strongly heating cubic MNP suspension, NC3 (*d*_TEM_ = 18 ± 1 nm, *d*_hyd_ 26.0 nm, PDI 0.04) in the Fe concentration range from 27 to 109 mM. The conditions used to synthesize NC3 were identical, in so far as is possible, to those for NC1 and NC2. The initial heating rates were found to increase in proportion to concentration, with an average SAR of 97 W g^–1^ (std. dev. of 1 W g^–1^ for the four concentrations) obtained, Figure 2b. This demonstrates robustness in extraction of the initial slope and that there is no particle aggregation in this range. The effect of the thermal probe location was also varied from 0 to 15 mm from the bottom of the eppendorf tube, at Fe concentration 109 mM, and the SAR values remained within 5% of the average. This demonstrates that the suspensions are homogeneous and there are no convective patterns. Hence, the huge variability in SAR is real and, given the full dispersion, it must arise due to differences in the internal structure of the cubic MNPs and how these alter the moment magnitude and/or dynamics.

**Figure 3:**
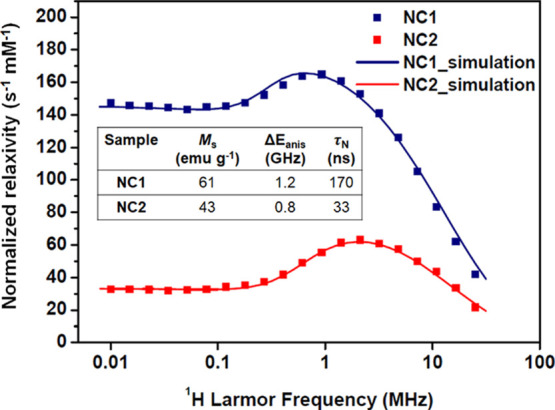
FFC-NMR profiles (*r*_1_ as a function of ν_L_) recorded at 25 °C for NC1 and NC2 suspensions in heptane. The Fe concentrations were 1.0 and 0.9 mM, respectively. The solid lines show the simulated profiles for which the parameters are indicated in the inset table.

The magnetic properties of the suspensions were further evaluated by measuring the spin–lattice relaxivities (*r*_1_) as a function of ^1^H Larmor frequency, ν_L_ (and hence field strength), by fast field-cycling NMR relaxometry.^26^ The FFC-NMR profiles obtained are commonly used for evaluating MRI contrast efficacy and for interrogating the moment dynamics.^27,28^ Experimental profiles obtained for NC1 and NC2 are shown in Figure 3, along with profiles simulated using the accepted SPM model^26^ that satisfactorily reproduce the experimental data. Note that these measurements were performed in the low millimolar Fe concentration range in which DLS demonstrates full dispersion.

The profile for NC2 (low SAR) shows the characteristic response of superparamagnetic MNP suspensions, with decreasing *r*_1_ in the high-frequency (clinical MRI) range, a mid-frequency maximum with *r*_1_ decreasing substantially to a low-frequency plateau, which are as expected for superparamagnetic MNPs in suspension. This observation further confirms full particle dispersion. Profiles have been previously described for water^26^ and heptane suspensions^29^ of spherical MNPs; this is, to the best of our knowledge, the first report of the profile for nanocube dispersions. The low-frequency relaxation is known to be dominated by the Néel process (moment reorientation in the magnetocrystalline field), and the high frequency part by the particles’ Brownian motion (as in this range the moments are locked to the external field). It is interesting that despite the distinct cubic shape, the profile is so similar to those recorded for spherical MNPs.^23^ This demonstrates that the Brownian correlation time, τ_B_, and the Néel correlation time, τ_N_, are comparable to those for similarly sized spheres. Superparamagnetism is generally associated with closed hysteresis loops under DC-field conditions at room temperature. In AC-fields, the delay between reversal of the applied field direction and dynamic realignment of the MNP moments results in heat dissipation.^27^ Longer delays correspond to a greater area enclosed by the AC-hysteresis loop, which is proportional to the SAR.^30^ Our interpretation is that for NC2, τ_N_ is too short (motion too fast) for there to be a significant SAR contribution at ν_AC_ 535 kHz.

For NC1 on the other hand, while the high frequency part of the profile converges toward that of NC2 (which has identical *d*_hyd_), as expected, below 10 MHz, the profiles differ substantially due to increased *τ*_N_ associated with greater internal magnetocrystalline anisotropy energy, Δ*E*_anis_, i.e., higher average barrier to moment reorientation within the cubes. Increased low-frequency *r*_1_ is well documented and is usually associated with the formation of clusters (and hence dipolar interactions) that retard the moment dynamics,^27,28,31^ although that is not the case here. The *r*_1_ value of 147 s^–1^ mM^–1^ measured at 0.01 MHz is extraordinarily high for a fully dispersed, and field-stable, suspension of identical *d*_hyd_ to NC2. The high relaxivity can be unambiguously attributed to slower average moment reorientation in NC1, the longer τ_N_ at room temperature also gives rise to increased hysteresis (open loops) and a high SAR at 535 kHz.

Simulations were undertaken using the accepted SPM model developed by Muller and co-workers.^26^ Initial attempts to simulate the experimental profiles assuming a nonmagnetic surface layer of thickness 0.5 nm (comprising pinned or canted spins) did not reproduce the experimental *r*_1_ maximum, or high frequency response, irrespective of the other parameters used. It was found that using the TEM edge lengths reproduced the experimental profiles (in particular, the frequency of the *r*_1_ maxima), and the other parameters could then be varied to obtain the best agreement, Figure 3. The NMR size obtained reflects the accessible surface sensed by the solvent molecules during the MNP encounter that drives ^1^H relaxation, rather than the core or magnetic size. We have previously shown the NMR size to be slightly larger than the core size for glycopeptide-stabilized spheres in H_2_O^18^ and for oleyl-stabilized spheres in heptane,^29^ suggesting the ligands prevent close access. The necessity of using the edge length for oleyl-stabilized cubes may reflect geometric issues, i.e., the distribution of distances of the closest approach. The simulations were found to be highly sensitive to the saturation magnetization (*M*_s_), adjusting it after fixing the size was the most important factor in reproducing the experimental profile, so the uncertainty in this parameter is small. The *M*_s_ and τ_N_ values obtained from the simulations are consistent with expectation for MNPs in this size range^27^ and with the XRD and microstructural analysis below. The Δ*E*_anis_ and τ_N_ values were then adjusted to provide almost perfect agreement in the mid-frequency range. The success of the simulations shows that the different chemical environments of the ^1^H in heptane have no measurable effect on the relaxivity.

The SPM model has several assumptions (including a uniaxial magnetocrystalline field and perfect size monodispersity), so while interpretation of the trends for the suspensions may be instructive, the absolute values of the extracted parameters should be viewed with some caution. Nevertheless, it is interesting that the simulations show *M*_s_ values in the expected range and with a much higher value, by a factor of 1.4, for NC1 and also higher τ_N_ (and Δ*E*_anis_) for this suspension, as is also shown by the SAR analysis. Given the similarity in size (the “cubic” nanocrystal volume of NC1 is only ca. 19% higher than NC2), the significantly higher *M*_s_ for NC1 must be associated with better crystallinity, which is confirmed by the microstructural analysis presented below.

For cubic MNPs, the magnetocrystalline easy axis is generally accepted to be along the [111] direction, pointing toward a corner.^32^ On careful inspection of TEM images recorded for NC1 and NC2, Figure 1b, sharper corners are evident for NC2. These would be expected to generate deeper local energy minima for the moment (and hence longer τ_N_) for NC2; however, the experiments and simulations suggest otherwise. We suggest that NC2 defects in the crystals prevent orientation of the moment toward the corners (however sharp), effectively reducing Δ*E*_anis_ and τ_N_. While this explanation would be equally applicable for any other orientation of the easy axis (toward an edge or face), the divergence in the hyperthermic and relaxometric properties for the two suspensions strongly suggest lattice distortions as the underlying cause for the low SAR and *r*_1_ in NC2.

**Figure 4:**
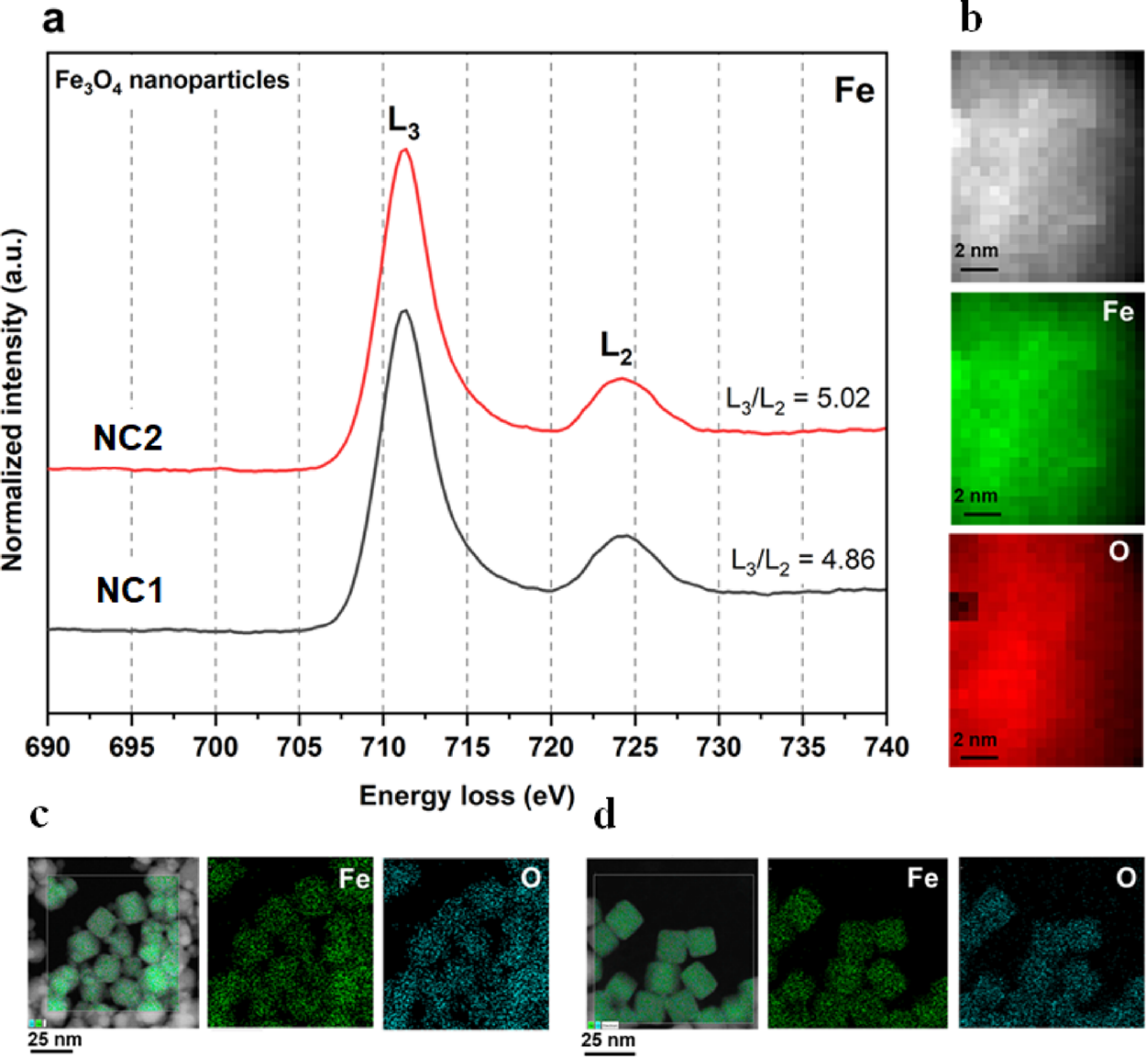
STEM-EELS and STEM-EDS analysis of cubic iron oxide nanoparticles obtained from NC1 and NC2 confirming the uniform elemental composition. (a) STEM-EELS analysis (0.3 eV/ch) representing the iron L_2,3_ ionization edge of the spinel phase of NC1 and NC2. (b) STEM-EELS localized nanometer scale elemental mapping. (c) STEM-EDS mapping of nanocubes from NC1. (d) STEM-EDS mapping of nanocubes from NC2, indicating the uniform presence of iron and oxygen for both samples.

Microstructural analysis of NC1 and NC2 was undertaken, focusing first on the sample’s chemical composition. Iron oxide MNPs are typically composed of magnetite (Fe_3_O_4_), maghemite (γ-Fe_2_O_3_), or intermediate phases sometimes associated with a more oxidized outer layer. Oxidation can progress after extended exposure to air,^33^ which can lead to changes in the magnetic responses.^34^ Oxidation state and crystal structure analysis were performed using STEM and HR-TEM. STEM-EELS and STEM-EDS were used to evaluate the elemental composition and chemical oxidation states of cubic nanoparticles, the results for NC1 and NC2 are shown in Figure 4.

Figure 4a shows the STEM-EELS chemical analysis representing the L_2,3_ ionization edges of Fe as acquired for the cubic MNPs. For transition metals, L_2,3_ ionization edges appear due to the transitions of electrons from the 2p state to the unoccupied 3d state.^35^ Ionization edges L_3_ and L_2_ were observed due to transitions from 2p_3/2_ and 2p_1/2_ core states, respectively, to unoccupied 3d states localized in Fe species.^36^ The integrated intensity ratio of white lines (L_3_/L_2_) can be correlated with the Fe valence state. The Fe L_2,3_ ionization edges of both cubic MNP suspensions confirmed the characteristic energy difference Δ*E*(L_2_ – L_3_) of 12.93 eV corresponding to iron oxide.^37^ Furthermore, the L_3_/L_2_ was evaluated as 4.86 for NC1 and 5.02 for NC2, which correspond to magnetite.^37,38^ The absence of a low-frequency feature (prepeak) for the L_3_ edge and of a high-frequency feature (postpeak) for the L_2_ edge, which are characteristic features of maghemite,^39^ confirm the predominance of magnetite in both samples. The Fe_3_O_4_ spinel crystal structure consists of both Fe^2+^ and Fe^3+^ oxidation states with possible tetrahedral (Fe^3+^) and octahedral sites (Fe^2+^ and Fe^3+^).^40^Figure 4b shows the STEM-EELS elemental mapping localized on the nanometer scale confirming the uniform presence of iron and oxygen. Figure 4c,d shows the complementary STEM-EDS elemental mapping of cubic MNPs from NC1 and NC2, respectively. Overall, the results indicate that the variation in the magnetic properties of NC1 and NC2 is not associated with variation in the chemical oxidation states. Both batches of cubic MNPs possessed identical chemical composition associated with Fe_3_O_4_.

The microstructural characteristics of NC1 and NC2 were evaluated using SAED bulk characterization. Detailed analysis of SAED patterns is shown in Figure S1a,b for NC1 and NC2, respectively. The spinel Fe_3_O_4_ phase was confirmed with characteristic lattice planes (202), (222), (400), (242), (333), and (404) observed having *d*-spacings of 2.95, 2.47, 2.07, 1.69, 1.60, and 1.48 Å, respectively, for NC1. Similarly, for NC2, the characteristic lattice planes (202), (311), (400), (242), and (404), associated with the spinel Fe_3_O_4_ phase, can be observed. However, the diffraction ring associated with the (333) lattice plane was absent in the SAED pattern of NC2. XRD bulk analyses for NC1 and NC2 are shown in Figure S1c. The intensities of the (202), (311̅), and (400) lattice planes corresponding to *d*-spacings of 2.98, 2.54, and 2.10 Å, respectively, are lower for NC2 than NC1. Using the Scherrer equation, the evaluated crystallite sizes for NC1 and NC2 are 12.49 and 7.12 nm, respectively. For cubic MNPs, a shape factor of 0.94 was considered for calculating crystallite size.^41^ The calculated FWHM from the (311̅) highest intensity XRD peaks are 0.012175 and 0.0213173 radians for NC1 and NC2, respectively. These results further validate the interpretation of the FFC-NMR results and are complemented by HR-TEM analysis that follows, suggesting greater prevalence of lattice defects in NC2.

**Figure 5:**
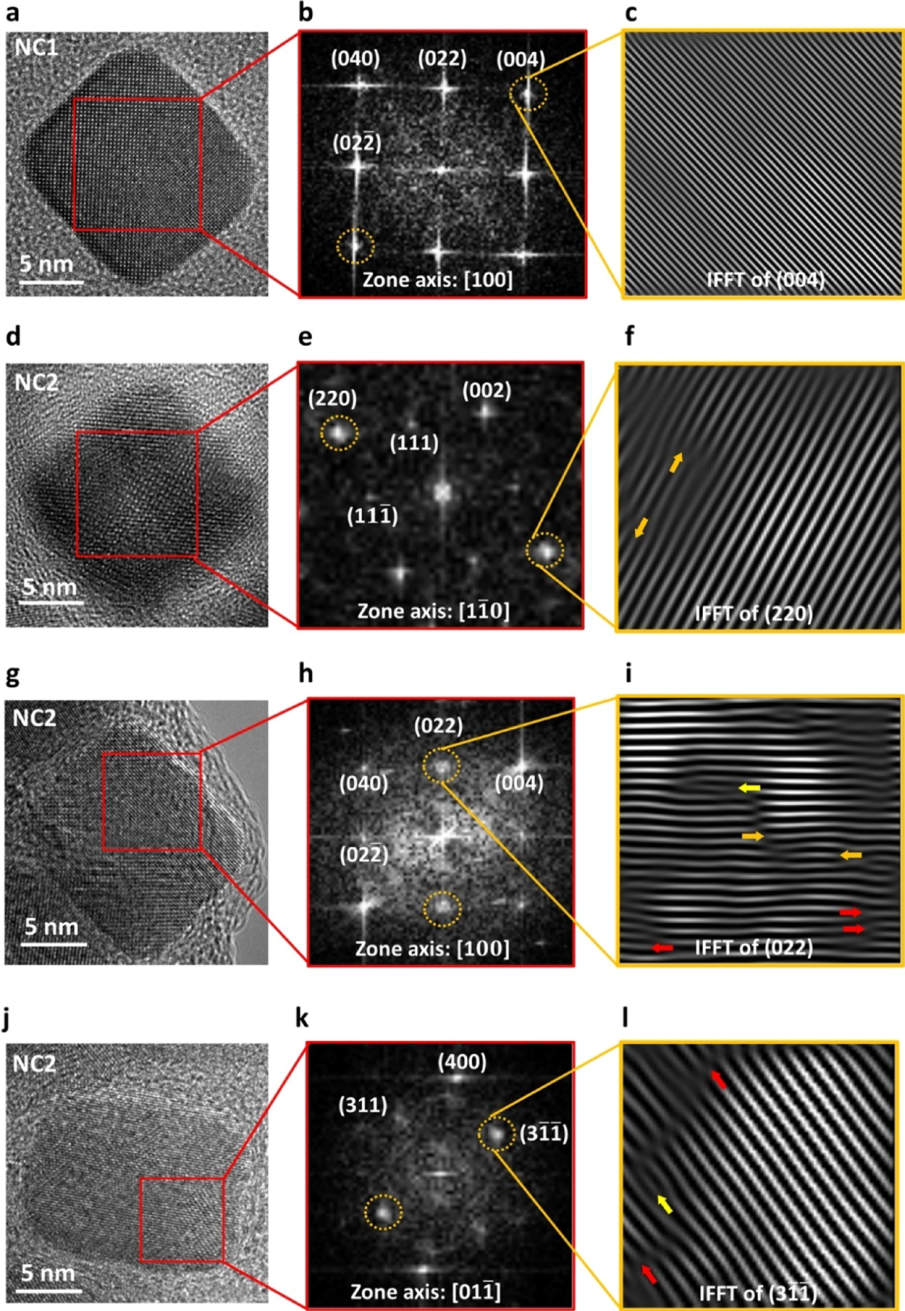
High-resolution TEM analysis of cubic MNPs from NC1 (a–c) and NC2 (d–l) demonstrating greater prevalence of lattice distortions in NC2. HR-TEM micrographs (a,d,g,j) of representative cubic MNPs from NC1 and NC2. The corresponding FFT patterns acquired from the localized crystal regions indicating the lattice planes along [100], [11̅0], [100], and [011̅] zone axes are represented in panels (b,e,h,k), respectively. (c) Inverse-FFT acquired from the (004) lattice plane confirming the absence of lattice dislocations in the representative NC1 crystal. Panels (f,i,l) showing inverse-FFT acquired from (220), (022), and (31̅1̅) major lattice planes, respectively, confirm the presence of planar lattice dislocations in the representative NC2 crystals. Orange arrows indicate interstitial extra half planes, yellow arrows indicate specifically extrinsic stacking faults, and red arrows indicate intrinsic stacking faults.

The lattice distortions indicated by the XRD analysis for NC2 were further investigated using HR-TEM. Figure 5 shows HR-TEM and associated fast Fourier transform (FFT) and inverse-FFT (IFFT) analyses for representative NC1 and NC2 MNPs. Figure 5a shows the HR-TEM micrograph of a representative NC1 particle; the corresponding FFT analysis from the identified region is shown in Figure 5b indicating lattice planes (040), (022), (004), and (002̅) associated with 2.09, 2.98, 2.09, and 2.98 Å *d*-spacings, respectively, in the [100] zone axis. Figure 5c shows the IFFT pattern of the (004) lattice plane confirming the absence of lattice dislocations.

Figure 5d shows the HR-TEM micrograph of a representative particle from NC2. The corresponding FFT analysis from the identified region is shown in Figure 5e indicating lattice planes (220), (002), (111), and (111̅) associated with 2.98, 4.23, 4.98, and 4.98 Å *d*-spacings, respectively, in the [11̅0] zone axis. Figure 5g shows the HR-TEM micrograph of another NC2 particle, and the FFT analysis, Figure 5h, shows lattice planes (040), (022), (004), and (002̅) associated with 2.09, 2.98, 2.09, and 2.98 Å *d*-spacings, respectively, in the [100] zone axis. Figure 5j shows the HR-TEM micrograph of a third representative NC2 crystal. The FFT analysis, Figure 5k, indicates lattice planes (400), (311), and (31̅1̅), in this case, associated with 2.12, 2.57, and 2.57 Å *d*-spacings, respectively, in the [011̅] zone axis. Figure 5f,i,l represents IFFT patterns of (220), (022), and (31̅1̅) lattice planes, respectively, confirming the presence of stacking faults. These are planar defects which can appear extrinsically, due to the inclusion of an additional atomic layer, or intrinsically, due to a missing intermediate atomic layer.^42^ Such stacking faults can originate during crystal growth from point defects or localized dislocations.^43^ Stacking fault defects associated with partial dislocations play a critical role in cross-slip and gliding of dislocations.^44^ Extra half planes apparent in Figure 5f,i, represented by orange arrows, are clear evidence of edge dislocations indicating interstitial-type stacking faults.^45^ In Figure 5i,l extrinsic and intrinsic stacking faults are represented by yellow and red arrows, respectively. Furthermore, analysis of additional NC2 crystals confirmed the presence of a twin boundary and an edge dislocation, see Figure S2a,b, respectively. Formation of twin boundaries is highly sensitive to the chemical environment and events during the MNP synthesis.^46^ Edge dislocations can induce local strain fields in the lattice.^47^ It is clear that distinct defects are present in NC2 cubic MNPs.

We suggest that these localized defects adversely affect the moments’ magnitude and dynamics, resulting in poor collective magnetic characteristics, as observed for NC2. Similar observations were reported by Pellegrino and co-workers^11^ based on microstructural analysis of a single low SAR spherical iron oxide MNP. The comparative structural analysis presented here along with the evaluation of both the MRI and hyperthermic responses, for samples that are identical in all other respects, confirms the role of crystal integrity. For the low SAR suspension (NC2), crystal defects prevent moment orientation toward the corners, and clearly, the particles have reduced magnetic volume and hence lower magnetization and lower magnetocrystalline anisotropy, giving fast moment reorientation, as determined by FFC-NMR.

**Figure 6:**
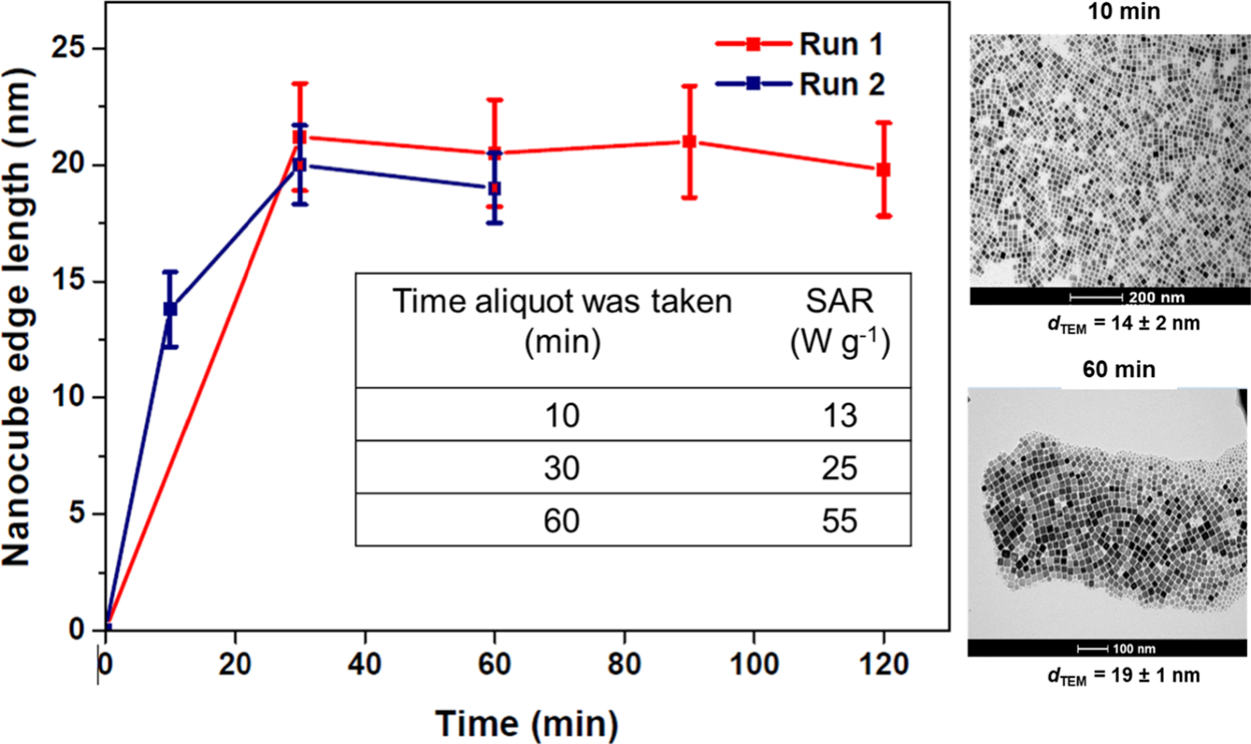
Temporal size evolution of cubic MNPs synthesized using FeOl in squalene with L/P = 1. Aliquots were drawn at the times indicated after the onset of reflux. All the aliquots were stabilized in heptane for SAR measurement and TEM preparation. The *d*_TEM_ values given are the sample (*n* = 100) averages. Inset table, the SAR measurements for Run 2.

Finally, the causes of batch-to-batch variability in crystal integrity were evaluated by studying the evolution of a suspension’s characteristics during synthesis. Multiple samples were drawn from a reaction at different times for TEM and hyperthermia analysis, see Figure 6. Time zero is defined as the start of reflux. It is clear that within 10 min, good cubic morphology was achieved, and the *d*_TEM_ value was already found to be 14 ± 1 nm with an aspect ratio of 1.00 ± 0.02. Hence, the system was in the growth phase by this time and the measured SAR was low. Over the full hour of the reaction, the size increased to 19 ± 2 nm and the SAR to 55 W g^–1^. Hence, this is a reasonably high heating sample, akin to NC1 albeit with the efficacy somewhat suppressed, most likely due to the disturbance caused by taking aliquots. It is clear that the SAR evolves throughout the growth phase due primarily, we suggest, to improving crystallinity. However, as a subpopulation of small particles is evident at 60 min, it is not possible to eliminate the possibility of a significant number of smaller nonheating particles contributing to the low SAR for the aliquot experiment. No such subpopulations are present for NC1 and NC2, for which we suggest that differences in crystallinity give rise to the hugely differing SAR values.

It was also found that in this case, extending the reaction for a greater time did not further improve the SAR. It can be concluded that high SAR suspensions are associated with successful annealing due to elevated temperature (∼285 °C) that relieves magnetic frustration. It is not possible to demonstrate whether the partially annealed defects arise from predominantly twinned nuclei, or if all particles grow only by monomer addition. In either case the outcome is unpredictable, its extent varies from reaction to reaction despite identical synthesis conditions for NC1 and NC2. Therefore, to tailor the magnetic characteristics of the MNPs, it would be interesting to investigate the stochasticity associated with the MNP growth phase and how it influences crystal structure integrity.

## Conclusions

Clear differences in the microstructural characteristics of the nanocube samples pinpoint better crystallinity and the absence of defects as responsible for high hyperthermic and relaxometric efficacies for fully dispersed nanocubes. Interestingly, the presence of defects is found to be more important than the sharpness of the corners or surface layer effects in determining the depth of the magnetocrystalline energy minima. Samples with higher prevalence of planar lattice defects are found to have both lower *M*_s_ and τ_N_. These two effects, which are revealed by the difference in low-frequency *r*_1_, combine to greatly reduce the SAR for otherwise identical nanocrystals. Hence, this work contributes to understanding of how crystallinity determines both the strength and the dynamics of the magnetic moments and demonstrates the importance of defect-free crystals. It also identifies that exceptionally high low-frequency *r*_1_ is possible for relatively small defect-free fully dispersed nanocubes. Such materials may have applications for *T*_1_-weighted imaging in ultralow field MRI. It is anticipated that with the emerging possibility of identifying easy axes for individual crystals, through magnetic force microscopy,^48^ new possibilities for understanding the effect of different types of defects on the ensemble magnetic properties may soon be realized. Finally, the potential of process optimization and crystal engineering of cubic MNPs, and in particular of the conditions during the growth phase, is identified as key for realizing higher and more consistently reproducible hyperthermic and relaxometric responses.

## Supporting Information Available

Crystal structure analysis of NC1 and NC2 based on SAED and XRD measurements and additional high-resolution TEM analysis of cubic MNPs from NC2 (PDF)

## Abbreviations

AAS: Atomic absorption spectroscopy
*d*_hyd_: Average hydrodynamic size
*d*_TEM_: Average TEM size
τ_B_: Brownian correlation time
EDS: Energy-dispersive X-ray spectroscopy
EELS: Electron energy loss spectroscopy
FFC-NMR: Fast-field-cycling nuclear magnetic resonance
FFT: Fast Fourier transform
HR-TEM: High-resolution transmission electron microscopy
L_2,3_: Ionization edges of iron (white lines)
FeOl: Iron oleate
MNP: Magnetic nanoparticle
MRI: Magnetic resonance imaging
Δ*E*_anis_: Magnetocrystalline anisotropy energy
τ_N_: Néel correlation time
PDI: Polydispersity index
STEM: Scanning transmission electron microscopy
SAED: Selected area electron diffraction
SAR: Specific absorption rate
*r*_1_: Spin–lattice relaxivity
XRD: X-ray diffraction.
