# Supplementary material for: Correlating Magnetic Hyperthermia and Magnetic Resonance Imaging Contrast Performance of Cubic Iron Oxide Nanoparticles with Crystal Structural Integrity
Source: Chem Mater. 2022 Aug 11;34(24):10801–10. doi: 10.1021/acs.chemmater.2c00708 (PMC9798828; doi:10.1021/acs.chemmater.2c00708)
Supplement: Supplementary file 1 — cm2c00708_si_001.pdf [file cm2c00708_si_001.pdf]

Correlating magnetic hyperthermia and magnetic resonance imaging contrast performance of cubic iron oxide nanoparticles with crystal structural integrity

Sameer D. Shingte [a], *Abhijit H. Phakatkar* [b], *Eoin McKiernan* [a], *Karina Nigoghossian* [a], *Steven Ferguson* [c], *Reza Shahbazian-Yassar* [d], *Dermot F. Brougham* [a,*]

[a] School of Chemistry, University College Dublin, Dublin, Ireland

[b] Department of Biomedical Engineering, University of Illinois at Chicago, Illinois, USA

[c] School of Chemical and Bioprocess Engineering, University College Dublin, Dublin, Ireland

[d] Department of Mechanical and Industrial Engineering, University of Illinois at Chicago, Illinois, USA

SUPPORTING INFORMATION

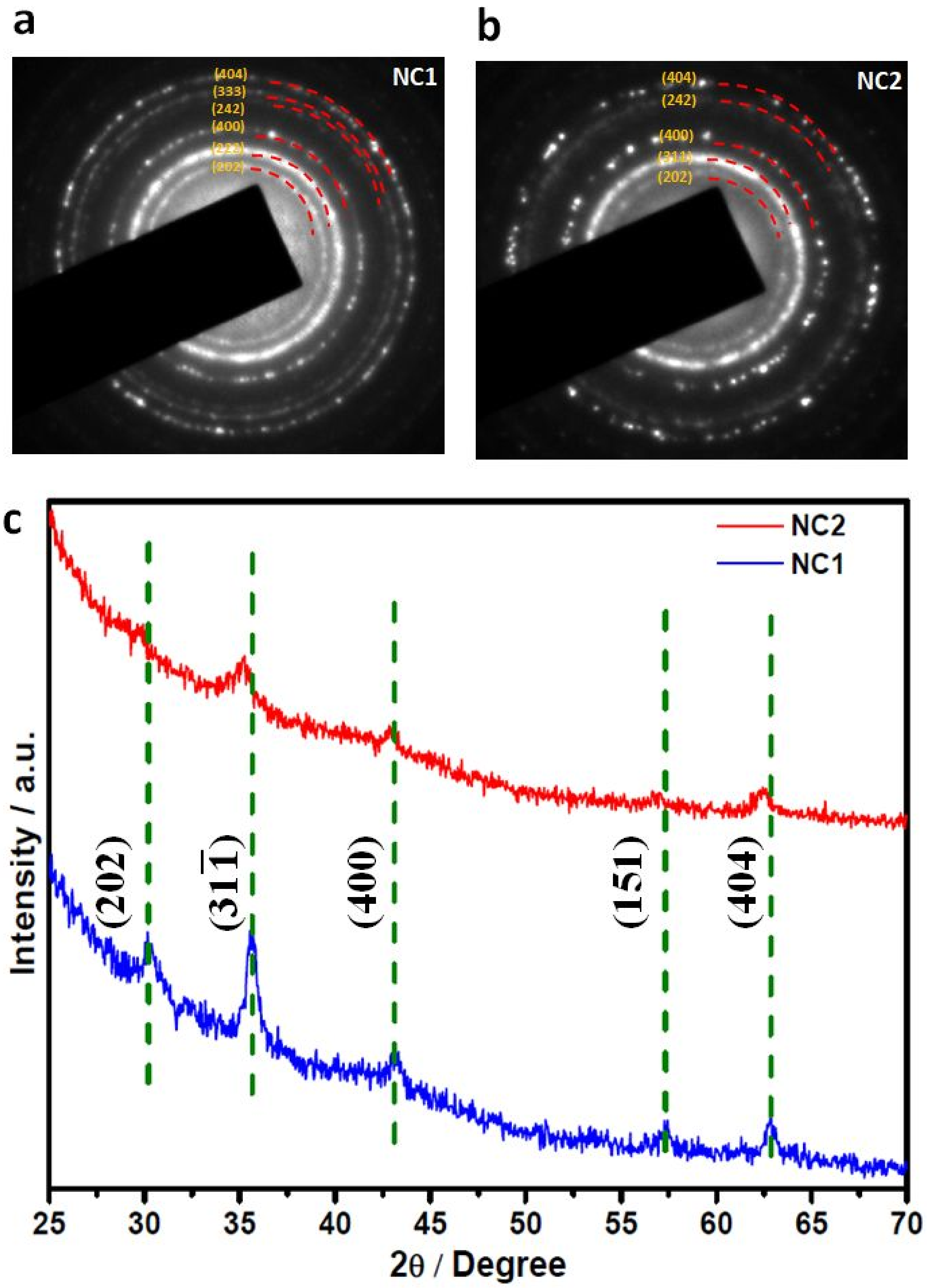

Figure S1. Crystal structure analysis. (a) SAED pattern of cubic MNPs from NC1 indicating the presence of the Fe_3O_4 spinel phase. (b) SAED pattern of cubic MNPs from NC2 indicating the presence of Fe_3O_4

spinel phase with poor crystallinity. (c) XRD analysis of cubic iron oxide MNPs from NC1 and NC2 highlighting the peak-shifts and differences in crystallinity.

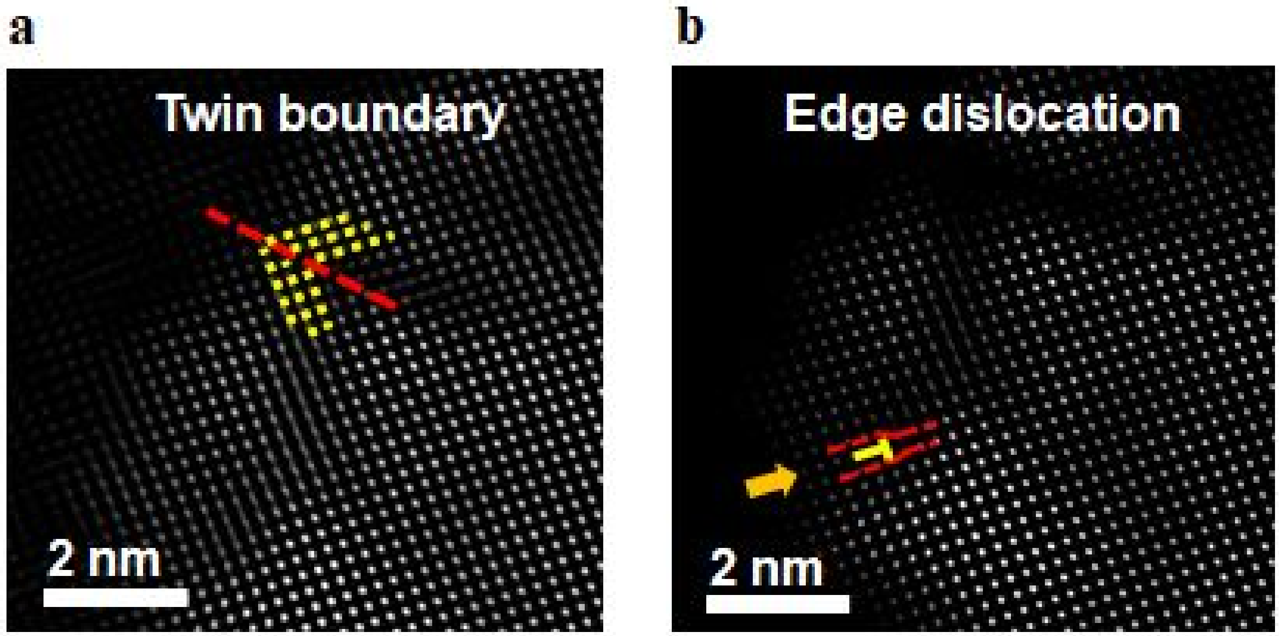

Figure S2. Additional high-resolution TEM analysis of cubic MNPs from NC2. (a) A twin boundary defect shown in IFFT acquired from a representative NC2 crystal. (b) An edge dislocation defect shown in IFFT acquired from the same representative NC2 crystal.
